# Binding of Ochratoxin A to a Urinary Globulin: A New Concept to Account for Gender Difference in Rat Nephrocarcinogenic Responses

**DOI:** 10.3390/ijms9050719

**Published:** 2008-05-08

**Authors:** Peter G. Mantle, Judit M. Nagy

**Affiliations:** 1 Centre for Environmental Policy, Imperial College London, London SW7 2AZ, UK; 2 Institute of Biomedical Engineering, Imperial College London, London SW7 2AZ, UK

**Keywords:** Ochratoxin A, mycotoxin, gel electrophoresis, immunoblotting, nephrotoxicity, female rat, renal carcinoma, α2u-globulin, evidence-based toxicology, risk assessment

## Abstract

SDS-gradient mini-gel electrophoresis and immunoblotting of urine of rats given ochratoxin A (OTA), showed OTA binding to an α2u-globulin. Perceived potential internalised delivery of OTA to proximal tubule epithelia by the carrier, specific only to adult male rats and augmenting other uptake mechanisms, suggests that some experimental nephrotoxicological data may not be appropriate for human risk assessment. Reexamination of female rat renal tumour histopathology of the NTP high dose OTA study showed all carcinomas were solitary, unilateral, microscopic and clinically insignificant at the 2-year end-stage. The novel concept, when consolidated further from our archived material, may moderate current perceptions of the human risk of traces of dietary OTA.

## 1. Introduction

The general toxicity of ochratoxin A (OTA) and its binding to plasma albumins has long been well known in several animal systems [1, 2, 3], but demonstration of its potent renal carcinogenicity [4], notable particularly in male rats, showed a sinister aspect that has since stimulated much research. Classification of OTA as “possibly carcinogenic to humans” [5], and its spasmodic trace occurrence in some foodstuffs of agricultural origin, has caused concern about food safety and impacts on food commodity trade. Binding of OTA with fairly high affinity to complex plasma proteins means that much of the circulating toxin is temporarily excluded from direct toxicological significance. Only the small proportion that is free in plasma at any one moment is available for renal excretion. However, variations in dissociation constants for plasma albumins in different types of animal will determine the extent to which free OTA is available for excretion in glomerular filtrate and accessible to whatever mechanisms facilitate absorption into tubule epithelia. Such absorption of toxin is hardly advantageous to an animal, but may be an inevitable consequence of one moiety of the OTA molecule being ‘recognisable’ as the essential amino acid phenylalanine, and the whole molecule potentially being an organic anion for which trans-membrane transporter proteins occur in renal tubules [3, 6].

Although glomerular filtration would seem to be the most obvious direct way in which a small organic xenobiotic such as OTA is eliminated via rat kidney, there have been several publications invoking activity of organic anion transporter proteins (OATs), demonstrable in tissue-cultured cells and in tissue cryosections and thought to function *in vivo* by transporting OTA from renal parenchyma interstitium into proximal tubule cells. However, it is difficult to envisage free OTA in circulating blood plasma leaking into renal interstitium and crossing the basement membrane more easily than to flow directly through the fenestrations of glomerular capillary endothelia. Arterial blood in kidney cortex flows primarily to glomeruli before returning as peritubular capillaries to collect vital low molecular weight nutrients scavenged from glomerular filtrate.

In the present context the important consideration is whether expression of OATs in adult rat kidney varies between sexes; notably OAT 1 and OAT 3, proposed as OTA transporters, was expressed similarly in kidney of pre-pubertal Sprague Dawley males and females [6] but OAT 2 mRNA expression increased significantly in females relative to males after 35 days old. In older adults OAT 1 was expressed a little more in males than females, and OAT 2 was expressed much more in females than males [7]. In other studies in adult Wistar rats [8], greater constitutive expression of OAT 1 and OAT 3 in male cortical tubule epithelia was illustrated, and shown to be influenced by androgens. However, over an OTA dose range of 50–500 μg/kg *per os* on alternate days for 10 days, lower doses up-regulated expression of OAT 1 and OAT 3 but higher doses down-regulated expression in cortical tubules [9]. Correspondingly, when compared with controls, no significant change in expression of genes associated with organic anion transporters was detected in kidney of male Fischer rats during the first year of continuously ingesting OTA-contaminated feed and potentially leading to carcinogenesis [10]; the dose was in the middle of the above range. Comparative studies on renal cortical homogenates from pig, mouse, rat and human did not reveal involvement of any common binding component, such as a known organic anion transporter protein [11]. Thus the role of cortical OATs in rat renal elimination and toxicology of traces of OTA that can cause renal cancer remains uncertain. Perhaps this explains why a recent review [12] makes only brief and unclear mention of the role of OATs in OTA elimination *in vivo*.

In any case, rather little free OTA is excreted directly in urine, recognisable elimination being mainly in the form of OTA's isocoumarin moiety, released by peptide cleavage of the toxin to give ochratoxin alpha. Some of these processes have been studied *in vitro*, but the pharmacodynamics of renal handling of traces of OTA in glomerular filtrates of animals ingesting traces of the toxin in feed remains unclear [13].

Another putative mechanism for rat gender difference in OTA renal carcinogenesis was proposed [14] involving different degrees of expression of some cytochrome P450 enzymes. However, the analyses were made on kidney from the 2-year end-point in an OTA carcinogenicity study [15] and it is not stated whether any of the organs contained neoplastic tissue. It is difficult to relate any differential enzymology findings in the kidneys of rather elderly and questionably carcinomatous rats to the situation in dynamic adults during the first year of life. The first year is the period during which dietary OTA exposure can programme sufficient tumour initiation and promotion for tumours to develop later in the second year [16]. Thus we conclude that there is yet no plausible mechanism to explain gender response differences seen in young adults.

Recent lifetime studies on rat renal tumours [16, 17 and our unpublished data] have generated material and experimental context for fresh study of the marked gender difference in carcinogenic response first emphasised in the classic NTP study [4]. Involvement of binding of OTA to androgen-dependent α2u-globulin, with consequent putative involvement of a specific α2u-globulin-nephropathy, exemplified by d-limonene [18], has been discounted in rat OTA nephrotoxicity though by indirect experiment [19], The finding has been generally accepted [20, 21, 22]. Alpha2u-nephropathy is typified by the binding of d-limonene so tightly in a pocket within the protein structure that, when absorbed into proximal tubule epithelia, the cells are unable to recycle the protein. Consequently, it accumulates. Cellular proliferation to replace consequent morbidity is thought to account for ensuing nephropathy. However, generalised application of this hypothesis has recently been questioned [23].

α2u-globulin, synthesised in male rat liver, is rapidly excreted via glomerular filtration and therefore has a very short half-life in plasma [18]. If dietary protein is not abundant the small proteins are reabsorbed rather efficiently into proximal tubule epithelium so that they remain rather cryptic and internalised in the male rat. However, on standard laboratory rat diet, there is less need for stringent protein economy and traces of globulins can be found in adult urine, allowing detection and recognition as in the present pilot study.

## 2. Materials and Methods

### Experimental urine samples

A male Fischer rat, age 14 months and 500 g body weight, was given a large dose of OTA (6 mg) dissolved in dilute sodium bicarbonate by oral gavage. The OTA was from the same preparative batch as was supplied to some partners in a recent EU-sponsored research programme and the quality of which had been independently verified [24]. Urine, collected into 1 ml 1% sodium azide in a metabolism cage overnight after 2 days and stored at −20ºC, was selected for analysis of response to this acutely toxic insult. Another male rat, a cross between a Sprague-Dawley female and a Fischer male, was selected because this hybrid avoids the inherent tendency of Fischers to develop a spontaneous mononuclear leukaemia, was given OTA as a 5 ppm dietary component (20 g feed; 100 μg OTA daily) for 250 days from pubescence. Similarly, urine was collected overnight in a metabolism cage for analysis of response to a well-tolerated chronic exposure, typical of that used for recent lifetime renal tumour studies [17]. Urine from a female litter-mate of the above Sprague-Dawley x Fischer hybrid, similarly given OTA, was also available.

A few μl of urine was collected a few hours after delivery by the supplier (Harlan) from each animal in two weight-matched groups of three pubescent Sprague-Dawley males from the same delivery group and of similar age but with weight in the ranges 180–184 g and 186–192 g. Urines in each group were combined for analysis. Urine was also collected from an adult male mouse (strain IL4/13K0), currently resident in the animal facility.

### Sample preparation and SDS gel electrophoresis

Urine samples from rats showing oligouria were concentrated ten-fold using the Micron ultracentrifugal filter units (Millipore, Watford, UK) following the manufacturer's instructions. We wished to ensure that sufficient α2u-globulin, likely to be occurring only in trace amount in urine and possibly including some molecules carrying OTA, could be available for electrophoretic resolution in the <1 µl applied to a mini-gel.

SDS gel electrophoresis and Western blotting was carried out using the GE Healthcare (Amersham, UK) Phast system. Protein samples obtained from the concentrator were diluted to the desired concentration in Phast gel loading dye and heated at 85ºC for 5 min. Then, 2.5 µl of each sample were loaded on 8–25% gradient precast Phast-gels. Gels ran for 20 min, according to the manufacturer's instructions, and were stained with Imperial colloid coomassie stain (Pierce, Rockford, IL, USA) and imaged by the Syngene (Cambridge, UK) Dyversity imaging system. Immunoblotting used OTA primary antibody purchased by special arrangement from R-Biopharm Rhone Ltd., Glasgow. The controls were male rat α2u-globulin (courtesy of Dr M. Dobrota, University of Surrey) diluted to an appropriate concentration, or α2u-globulin mixed with OTA of similar molarity, incubated at RT for 10 min and then loaded on the SDS page gel. The secondary antibody was Sigma anti-mouse (polyclonal). Western blots were visualised using the ECL plus kit from GE Healthcare (Amersham, UK) and imaged on the Syngene (Cambridge, UK) Dyversity imaging system.

## 3. Results

**Figure 1.**
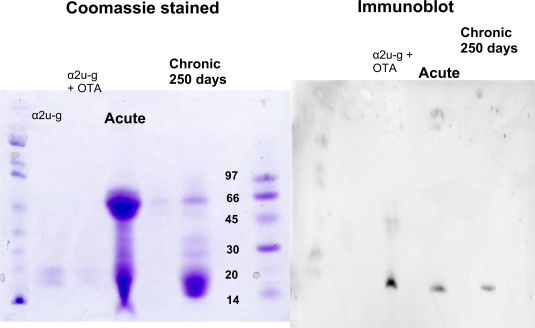
Matched gradient-gel electrophoretograms comparing resolution of urinary proteins of a mature Fischer male rat after acute OTA intoxication and a hybrid male rat after chronic OTA exposure with a reference sample of male rat α2u-globulins with and without added OTA. Left, stained with coomassie blue, right after immunoblotting with OTA antibody. Molecular marker in kDa.

**Figure 2.**
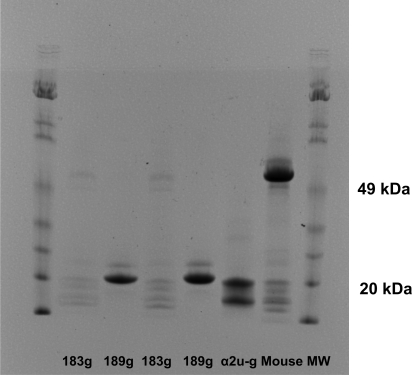
Coomassie-stained gradient-gel electrophoretic resolution of urinary proteins of male Sprague-Dawley rats (groups of three, mean ~183 g and ~189 g; ranges 180–184g and 186–192g) and of an adult male mouse (25 g).

Mini-gel electrophoresis and coomassie staining showed the two major isoforms of α2u-globulin in the reference sample (Figures 1 and 2), resolved into the 18.8 and 18.1 kDa compounds previously described [25, 26] and designated isoforms A and B, respectively. Molecular biology evidence showed [26] that in adult Sprague-Dawley and Fischer males the isoforms A and B are synthesised in a ratio of 1:4. Thus the mature adult hybrid male used in the present study is likely to have had similar synthetic potential and it is concluded that the two isoforms are present on the gel. In contrast, analysis of the urine of a female litter-mate, collected concurrently, revealed no evidence of the globulins (gel not shown), consistent with the well-established androgen dependence of gene expression for these proteins [17].

Mini-gel electrophoresis and immunoblotting with OTA antibody revealed binding of OTA to the reference sample of α2u-globulin after simple mixing of an aqueous solution of both components (Figure 1). Also, clear evidence of a similar association was evident in urine of the mature male rats given OTA either as a single acute dose or after many months of continuous ingestion (Figure 1). Incidentally, the urine from the rat given a large acute OTA dose also contained a notable content of a protein of ~50 kDa, partially reflecting the tubular epithelial damage from the acute insult that was evident also by an approximately 20-fold increase in total urinary protein and concomitant marked temporary glucosuria and haematuria. In contrast, the Sprague-Dawley x Fischer hybrid given dietary OTA showed no excessive proteinuria or other abnormality. However, the narrow resolution of the two main isoforms of α2u-globulin in the mini-gel limited definitive matching of the immunoblot with the coomassie-stained small proteins to identify which globulin isoform close to the 20 kDa molecular marker was involved.

First occurrence of the 18.8 kDa isoform of α2u-globulin in urine of pubescent male rats was shown to be during the 180–190 g transition in Sprague-Dawley males (Figure 2). Electrophoresis of urine from a mature male mouse also revealed a pair of proteins of a size similar to those of the rat α2u-globulins (Figure 2).

## 4. Discussion

From the present analyses, we perceive a role for probably just one α2u-globulin as an OTA-carrier protein, augmenting other OTA-uptake mechanisms in male rat nephrons, with effect increasing through puberty according to the well-established sharp increase in synthesis of the globulins in liver between 70 and 100 days of age [26].

By providing an exclusively male mechanism for enhanced rate of transfer of some circulating OTA to proximal tubules, the concept should result in relatively increased plasma OTA concentration in females if they lack any androgen-dependent mechanism for elimination. Conversely, females might sustain a lower OTA concentration in kidney during its temporary passage through the organ.

Notably, therefore, markedly higher plasma OTA concentration was measured in female adults (Sprague Dawley x Fischer hybrids) after 8 months of continuous dosing [27]. Also, Fischer female rats achieved higher plasma concentration following a single dose of OTA [28]. Even in Dark Agouti rats (constitutively with a much shorter plasma half-life than Fischers [27]), dietary OTA for 4 weeks resulted in a higher plasma concentration in females [31].

The concept of a specific OTA-delivery system augmenting proximal tubule exposure to the mycotoxin in the male rat could explain the greater histopathological response of male rats to OTA (1 mg) given once daily by oral gavage for 5 days than when the same amount was consumed in diet [29]. Relatively rapid delivery to proximal tubule epithelia of some of the OTA bound to the readily-filterable globulin could give a surge of toxic insult, whereas delivery to tubule epithelia from dietary absorption would give a less acute peak of daily insult. It is predicted that this would more readily cause nephropathy than the trickle feed of traces of free OTA in plasma that escapes from circulating blood by glomerular filtration during natural, more protracted, feed intake even with concomitant trickle feed *via* the globulin.

The present findings can similarly explain the significantly fewer male renal carcinomas in response to 2-year continuous dietary OTA [17] than to the intermittent oral gavage administration adopted in the NTP protocol [4], in spite of giving about twice the gavaged dose *via* diet.

There is apparently no human analogy of the male rat urinary globulins. Thus, extrapolation from dose-response data for experimental rat renal carcinoma to human risk assessment may only be appropriate for the female data, for which evidence is relatively sparse. OTA-attributed renal carcinomas in female rats have so far been limited, in the highest similar doses used, to three Fischer rats out of 50 in the NTP study, discovered only at the end stage [4, 30], and to a single animal out of 20 Lewis females, similarly discovered only at the end of a 2-year study [14, 15]. Further, in the latter study, there were no tumourous kidneys in 40 Dark Agouti females. However, karyomegalic nuclei were abundant in the cortico-medullary region of all Dark Agouti males, but only observed in ~10% of females. Applying the present concept, the Dark Agouti findings are consistent with gender differential of toxic insult to tubule epithelia, which can now be attributed to an androgen-dependent OTA-carrier protein. Detail on the single renal tumour in a Lewis female is obscure [15, 42, 43], but NTP has extensive archives.

**Figure 3.**
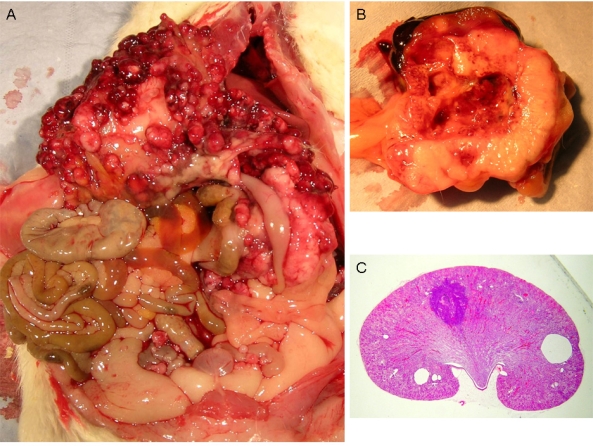
Comparative illustration of the contrast between maximum expression of renal carcinoma in response to chronic OTA exposure in male and female rats. A, Extensive carcinomatous nodules on abdominal serosal surfaces of male rat including diaphragm and prostate, but notably excluding bladder, metastatic from unilateral renal carcinoma (hidden); B, median section through one of the larger metastasising renal carcinomas illustrated by that of the animal in A, bearing two tangential patches of residual kidney tissue; C, LS kidney of female rat given the high OTA dose regime of the NTP study (Boorman 1989) showing the largest carcinoma, only discovered *in situ* at the end of the 2 year study (section courtesy of NTP Archives).

**Figure 4.**
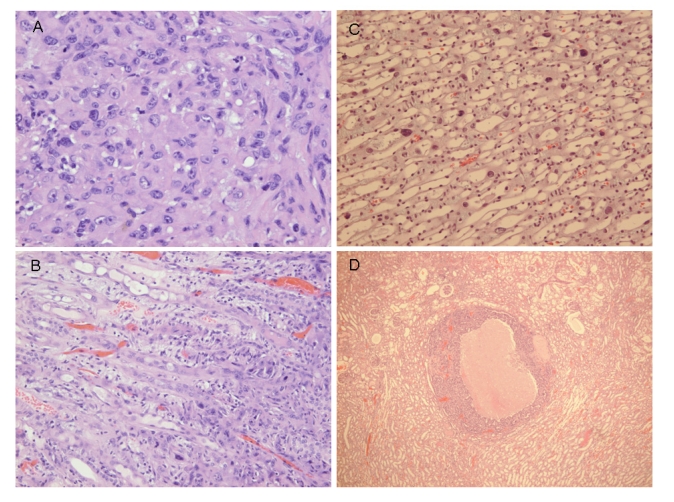
Micrographs of H & E sections of NTP female high OTA dose Fischer rats. A-C, detail of section in Figure 4C. A, representative carcinoma. B, carcinoma infiltrating renal medulla. C, prominent karyomegaly in the renal papilla. D, another example of a small renal carcinoma with necrotic centre, located at the corticomedullary junction.

Courtesy of NTP Archives, we have been able to review H & E sections of the tumourous kidneys of Fischer females from the OTA study and study all the benign and malignant hyperplasias. Comparison with the more obvious tumours in male rats, reported in the NTP study and also illustrated from recent experiments [16, 17], can be made from the example shown in Figure 3AB. In contrast, Figure 3C shows the magnitude of the largest (4mm diameter) carcinoma in female rats of the NTP study, and which did not even distort the kidney. Nevertheless, detailed histopathology shown in Figure 4A, B illustrates that there was typical disorganised carcinoma with many enlarged nuclei with prominent nucleoli, infiltrating surrounding renal parenchyma and reminiscent of that of male rat renal carcinomas in which DNA ploidy distribution has been measured as consistently aneuploid [16]. A surprising observation was extensive kayomegaly focused within the renal papilla of all female rat tumour-bearing kidneys of the high OTA dose group, where the section passed though the papilla (Figure 4C). This does not seem to have been reported before, but in the present context is interpreted as consequential in the female of a smaller proportion of excreted OTA, being transported into proximal tubule epithelia than in the male. Some free OTA in glomerular filtrate may then have been available to affect epithelia in the loop of Henle. Since the carcinomas were all unilateral and microscopic, and were discovered only at the end of the study, they were most unlikely at that stage to have significantly affected health. Another, even smaller carcinoma is shown in Figure 4D. None of the three carcinomas had metastasised.

**Figure 5.**
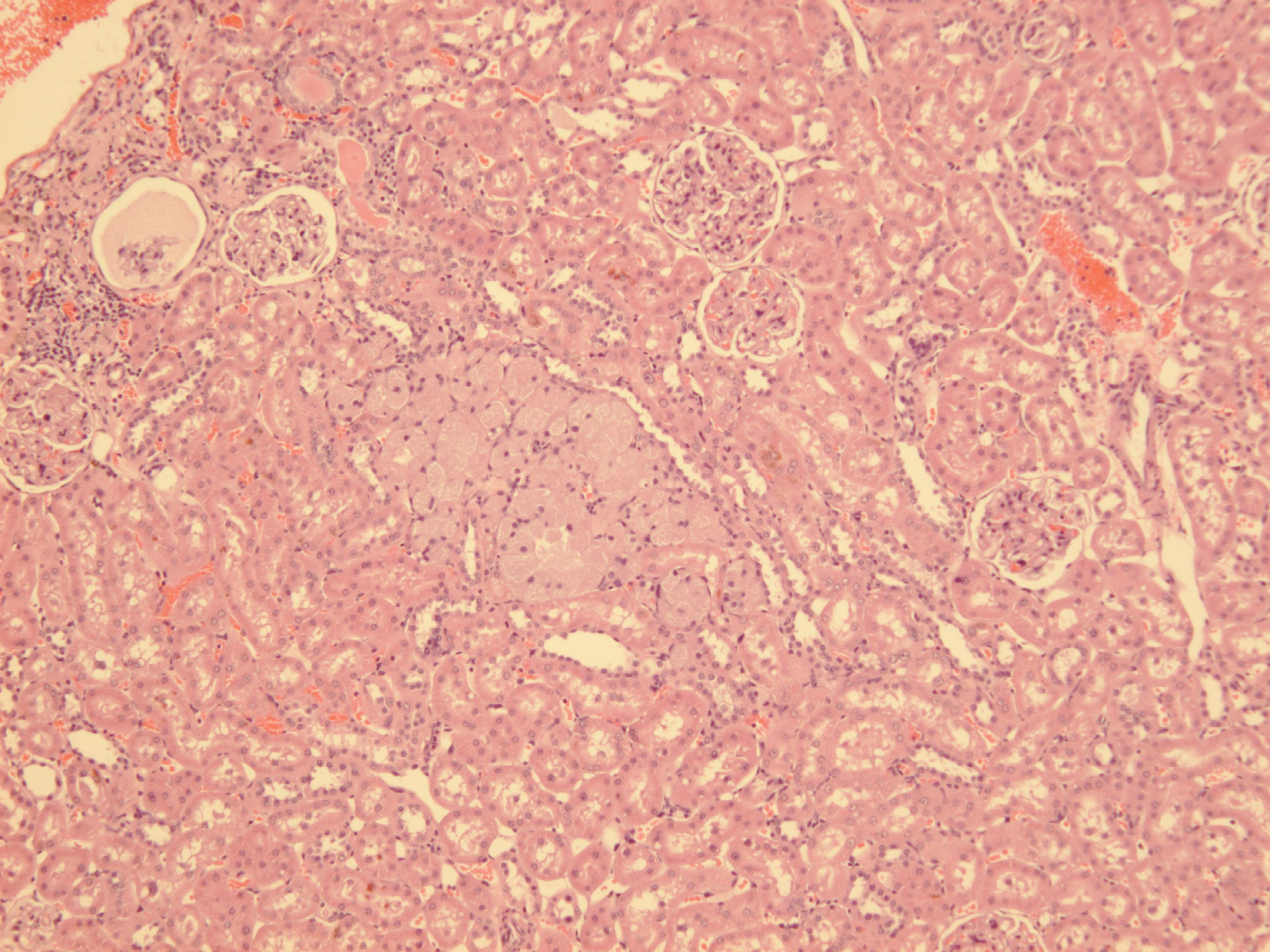
NTP female (105 weeks, high OTA). Focal adenoma sited just inside the innermost glomeruli. Karyomegalic nuclei are located between tumour and glomeruli.

Since illustration of female renal tumour histopathology ascribed to OTA has not previously been published, location of a small cortical neoplasm is illustrated in Figure 5 to show its origin close to innermost glomeruli, but karyomegalies typically caused by OTA are nearby.

**Figure 6.**
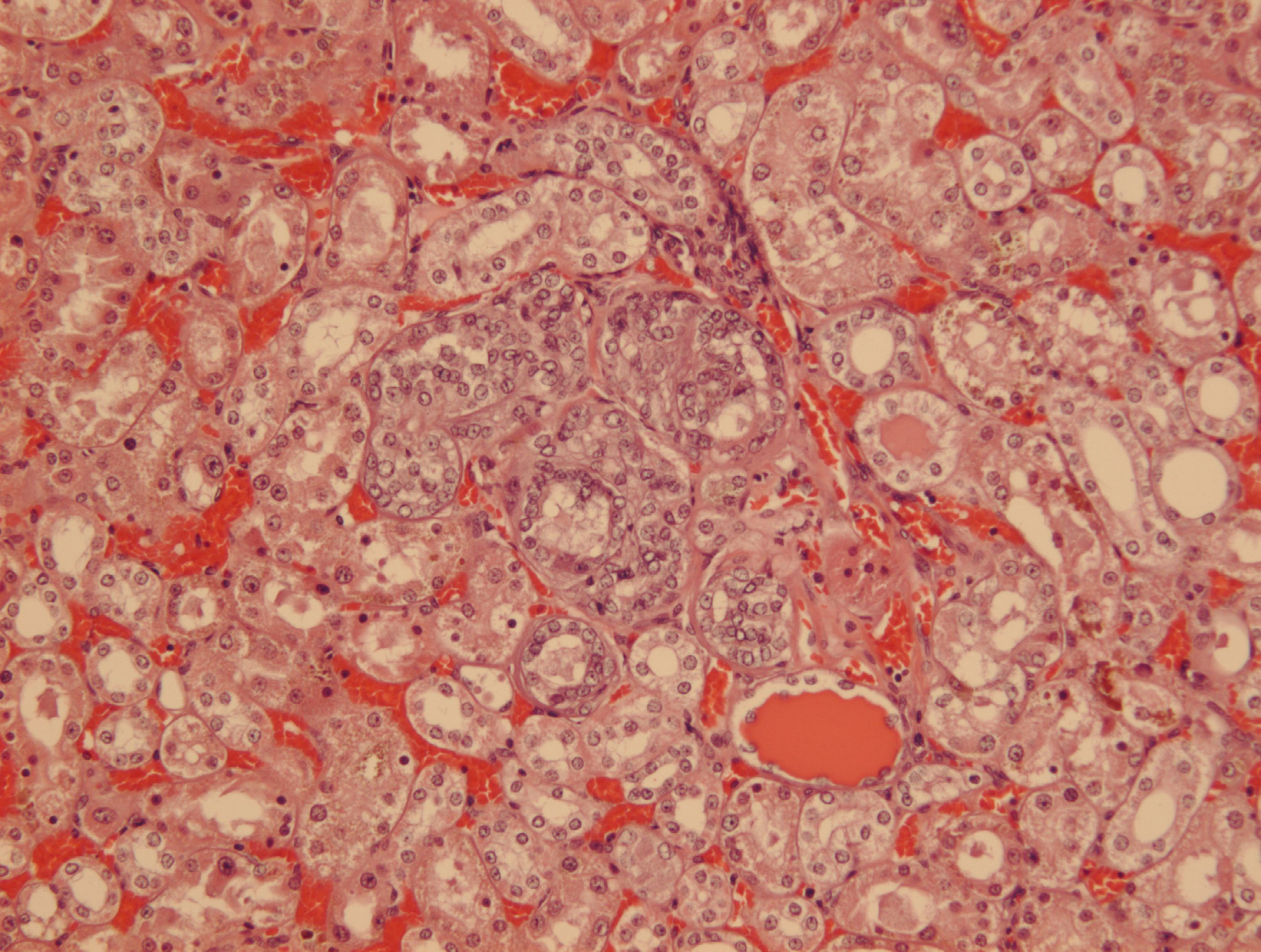
NTP female (91 weeks, high OTA). Small cluster of renal tubules with epithelial proliferation, constituting adenoma containing several karyomegalic nuclei.

**Figure 7.**
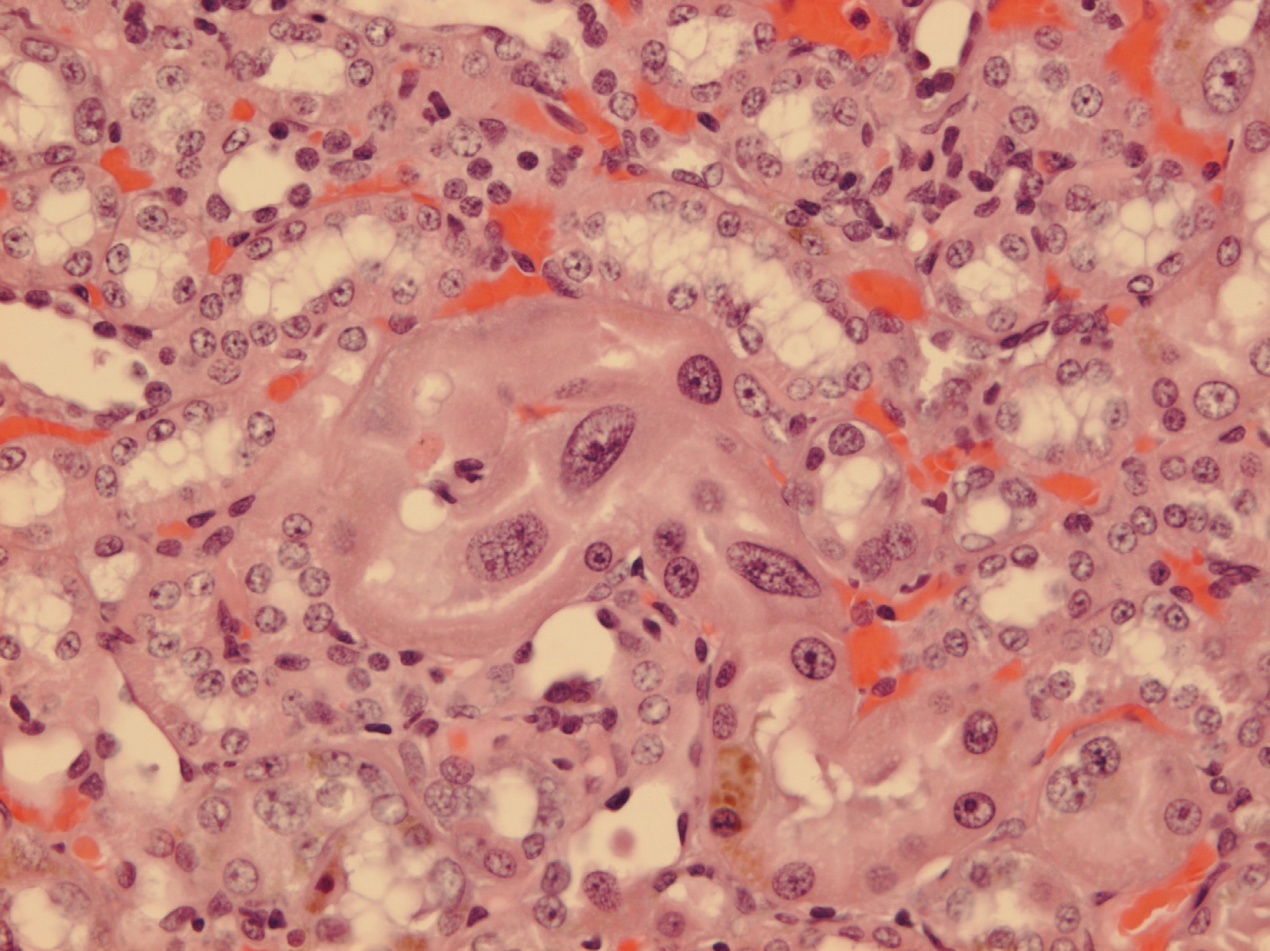
NTP female (104 weeks, high OTA). Prominent karyomegalic nuclei and mitosis in cytomegalic epithelium of inner-cortical tubule. Karyomegalic nuclei also in some surrounding tubules. Possibly a very early tumour.

**Figure 8.**
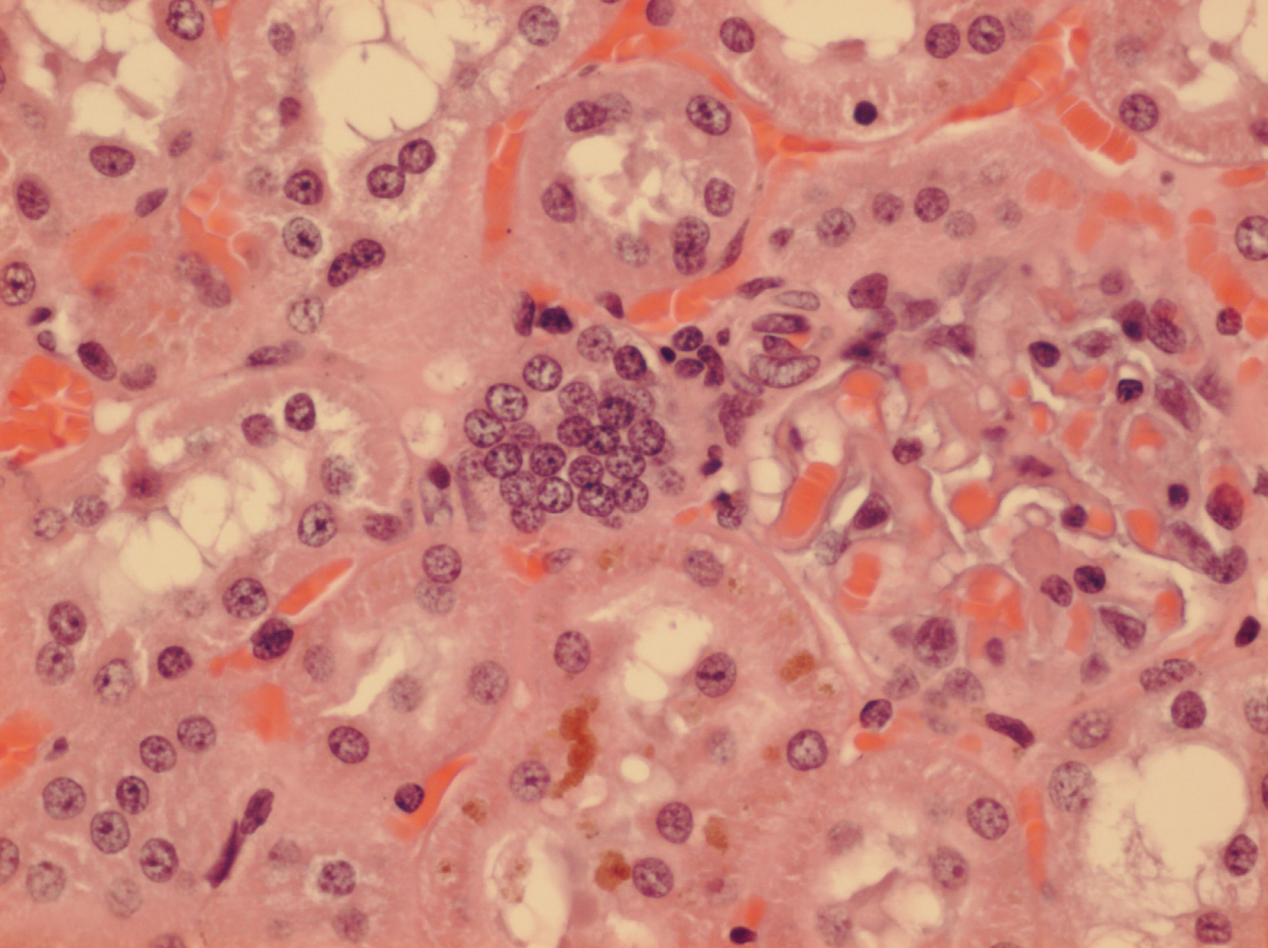
NTP female (104 weeks, high OTA). Nuclear proliferation in an inner-cortical tubule with karyomegalic nuclei in adjacent and nearby tubules. Probably an early adenoma.

Another example has karyomegalic nuclei already located amongst proliferating epithelial cells (Figure 6). The other few small solitary renal neoplasms in females at or near the 2 year endpoint of the NTP study seemed all to be located in the corticomedullary region (e.g. Figures 7 and 8).

To be inclusive of all published experimental female rat renal carcinoma data, the unilateral carcinoma discovered in the NTP mid-dose OTA group only at 46 weeks should be mentioned. It was unique in occurring very much earlier in life than all others, even in male rats in that and in all other subsequent lifetime studies which were only found from 75 weeks onward. Although spontaneous renal tumour is extremely rare even in Fischer females, caution should be exercised in placing undue significance on this single atypical finding.

In a very recent full-lifetime experiment with Dark Agouti male rats at Imperial College, more than 3 months of continuous exposure to dietary OTA (5 ppm) was necessary to cause renal cancer later in life; much of this period coincides with the period of maximum synthesis of α2u-globulin in liver [25, 26].

In determining the structure of the α2u-globulin in its complex with, for example, the hyaline drop inducer d-limonene, a binding cavity was observed, of dimensions appropriate for that small aromatic molecule [32]. In the present study, immunological recognition of OTA bound to an α2u-globulin isoform will have required access of the antibody to recognise the chlorinated toxin, utilising the antibody's high specificity for OTA as opposed to its deschloro-analogue ochratoxin B. However, the OTA-α2u-globulin binding is obviously not so strong as to make the complex function like those in which the globulin binds with several small molecules to cause typical α2u-globulin nephropathy [18]. Thus it is deduced that both intracellular release of the toxin and normal degradation of the globulin would occur within proximal tubules after uptake of the complex *in vivo*.

Predictably, intestinally-absorbed OTA travels within seconds to the liver where α2u-globulin is synthesised in the male rat and where binding to the virgin protein in blood can occur. The globulin has a very short half-life in blood, consequent on the hepatic product's opportunity for passage *via* ~50,000 glomeruli. Hence there will be rather little remaining subsequently in blood flowing to the intestines and in the hepatic portal vein. The known daily yield of hepatic α2u-globulin in a male rat [18] could predictably alone accommodate molar binding of ~1mg OTA, during its slow acquisition daily from feed. Rapid glomerular filtration could then deliver much of the bound OTA to target epithelia, complementing some of the small amount of freely-filtered OTA that is in competing dissociation equilibrium with serum albumins. Binding to the large serum proteins may vary between species and gender of experimental animals [11].

Gender difference in renal tumourigenic response to OTA in the mouse seems to be absolute, the male only being susceptible [33, 34], and unexplained. Mouse urinary protein(s) [e.g. 35] obviously deserve study to see if there is any similar binding with OTA. As with rats, focus should be on small proteins circulating in blood that, for reasons of physiological economy might be undetectable in urine because of efficient scavenge from glomerular filtrate by tubular epithelia.

The present new concept was first proposed at the 2007 IUPAC Mycotoxins Symposium (41), where also a poster presentation reported rather similar kinetics of OTA in plasma and kidney in prepubertal Fischer rats given a single oral dose of the toxin in dilute sodium bicarbonate [36]. In that report, toxin reached maximum plasma concentration within 2 hours and was maintained for at least another day. By using aqueous oral administration there was even more opportunity for any glomerulus-filterable, male carrier protein to operate than would be expected if OTA had been absorbed slowly from the vegetable oil used in both the NTP study and another [15]. The report [36] provides a firm basis for expecting no fundamental gender difference in renal pathology response to OTA in juveniles of the Fischer rat strain commonly used in NTP studies to detect worst-case sensitivities to xenobiotics. However, it would not be surprising that renal tumourigenic sensitivity of males and females could diverge if exposure to OTA occurs during puberty and subsequent sexual maturation. Therefore it is logical that the marked gender difference in both frequency of occurrence and in magnitude of expression of renal tumourigenesis (and most significantly in carcinogenesis) in response to OTA administered after puberty in well-replicated experiments [4, 15] could arise from a subtle change in physiological biochemistry caused in the male rat by hepatic synthesis of an androgen-dependent globulin to which OTA can bind.

Focus on the male rat renal carcinoma data from the NTP study has been a significant element in assessing the risk to human health of traces of OTA in some human foods. Such assessment remains hypothetical because OTA has not been proved to cause any human disease, although for some researchers it is a plausible aetiological factor for the Balkan endemic nephropathy and/or associated urothelial tumours [37]. However, it is important to note that the Balkan nephropathy tumours occur in the transitional cell tissues of the urinary tract and are not renal cell carcinomas. Nevertheless, silent early processes of urological carcinogenesis remain unclear, and extrapolation from the latter part of a standardised 2-year ‘lifetime’ of an experimental rat to any of the mainly-idiopathic human urinary tract tumours is particularly difficult. Experimental highest-dose/response findings for male rats in the NTP study recorded renal carcinomas in 60% of animals, which could be adjusted by Cox regression analysis to 77% to account for the unscheduled leukaemia mortalities in Fischer rats [4]. The data gave powerful evidence of OTA's malignant potential, and with the data from other dose groups has recently been seen as a perfect fit of an exponential dose-response relationship for a thresholded carcinogen based on thermodynamic principles in biology [38]. We calculate that the threshold thereby deduced from the NTP study data can be equated to a continuous dietary OTA content of ~250 ppb, and a recent study at Imperial College in Dark Agouti males (n=20) exposed for 2 years showed a zero-tumour threshold at 400 ppb. The thermodynamic principle should also apply to the NTP female data, modest as it is, thereby offering an even higher experimental threshold that might otherwise have been applied from male rat data to human risk assessment.

The aforementioned experimental findings relate to continuous 2-year ‘lifetime’ exposure to OTA, dosed either to maintain intake related to body weight in the NTP study [4] (although equivalent to a mean daily intake of ~150 μg/kg) or on a constant daily intake of ~100 μg in the recent study with the smaller Dark Agouti strain. The latter equates to about twice the NTP study dose on a body weight basis throughout most of adult life.

A recent review [39] based firmly on histopathological evidence from experimental rat tumours placed OTA in the category of ‘chemicals inducing renal tumours through direct interaction of the parent compound or metabolite with renal DNA’. Other indirect evidence has led some authors to conclude that OTA is not genotoxic [e.g. 44, 45] and this assumption was adopted in the latest EFSA review of OTA toxicity [46]. It has also been postulated that OTA's carcinogenicity in male rats is non-thresholded [47], but this seems an unfortunate deduction in view of the direct evidence from all lifetime rat studies, as far as their statistical power permits. However, since we are aware of recent direct structural data proving involvement of OTA in DNA adducts, we must view the present concept in the context of OTA being genotoxic however that term is defined. Further, a more severe attitude to OTA from the point of view of human risk assessment may arise when the structural data is published. Given that OTA directly damages rat renal DNA, but that incidence of DNA adducts falls below the limit of detection within a few weeks of toxic insult, it must not be assumed that repair is always total and perfect. Considering the relatively few genetic loci in which persistent damage is potentially tumourigenic, it is not surprising that several months of continuous genotoxic insults to renal tubule DNA are necessary to secure a critical genetic defect in one tubular cell in one kidney. Our unpublished data with Dark Agouti rats given continuous dietary exposure with OTA (5 ppm) showed that 6 months of a continuous barrage of renal tubule DNA damage was necessary to raise tumour probability above threshold. That the critical damage might be in one of the karyomegalic nuclei, that are well known to persist through life, has already been suggested [16]. The dose dependence of the incidence of renal carcinomas in male rats and in the frequency of bilateral tumour occurrence is consistent with dose dependent increase in probability of causing a critical genetic defect.

It is not clear from the JECFA and EFSA reports [20, 46] that panels considered qualitative rat tumour characteristics in addition to the quantitative dose-response incidence data. However, as is the case in human renal carcinoma, tumour proliferation stage has considerable bearing on the outcome of that morbidity. Amongst all experimental male rat renal carcinomas caused by OTA, there were several that were not only adversely affecting animal health by disabling a kidney but also by causing distant metastatic disease, of particular terminal consequence in lungs. In the case of the Fischer strain, leukaemia caused some renal tumours to be discovered earlier than their impact on animal health would otherwise have caused. Thus in rat strains that are less notable for their sensitivity to toxic agents, age distribution at tumour discovery could have been further extended into the ageing period and even more renal tumours could have become a health hazard. In contrast, the cryptic female carcinomas (exclusively from the NTP study), already set right at the end of the study, seemed to have little or no pathological significance within the standard 2-year experimental period that extended well into old age. Thus it would seem prudent to consider this in attempting to apply the female tumour data to human risk assessment, especially since there is no clear indication that OTA has ever caused human renal carcinoma.

In view of the present new concept of a glomerular-filterable male rat carrier protein for OTA, interpretation of all evidence in the experimental toxicology literature for OTA needs careful reappraisal if predictions for human exposure risk are sought on best available evidence [40]. Replication of gel electrophoretic data across more animals with different doses of OTA and periods of exposure was desirable, and would have been provided from our extensive archive of experimental materials if human resource had been available. Similarly, irrefutable structural proof of OTA binding to α2u-globulin would have been provided by mass spectrometric data from available instrumentation at Imperial College London if the necessary dedicated research assistance had been available. It is hoped that the present publication will stimulate debate and facilitate investment of the necessary resources to confirm the present concept. Of course the present concept needs consolidation from further proteonomic study, and our archived samples from *in vivo* experiments are already in place for analysis when other research resources become available.
